# CD157 Can Replace CD24 and CD14 in a Single-Tube Flow-Cytometric Assay to Detect Paroxysmal Nocturnal Hemoglobinuria (PNH) Clones on Both Neutrophils and Monocytes: A Prospective Study From North India

**DOI:** 10.7759/cureus.23965

**Published:** 2022-04-08

**Authors:** Roopam Deka, Hara P Pati, Dinesh Chandra, Prabhu Manivannan, Richa Chauhan, Seema Tyagi, Renu Saxena

**Affiliations:** 1 Oncopathology/Pathology/Hematopathology, Dr Bhubaneswar Borooah Cancer Institute, Guwahati, IND; 2 Hematology/Pathology, All India Institute of Medical Sciences, New Delhi, IND; 3 Hematology/Pathology/Hematopathology, Sanjay Gandhi Post Graduate Institute of Medical Sciences, Lucknow, IND; 4 Pathology/Hematopathology, Jawaharlal Institute of Postgraduate Medical Education & Research, Puducherry, IND; 5 Hematology/Pathology/Hematopathology, Sir Ganga Ram Hospital, New Delhi, IND; 6 Laboratory Medicine/Pathology, Medanta - The Medicity, Gurugram, IND

**Keywords:** paroxysmal nocturnal hemoglobinuria, pnh, flow cytometry, flaer, cd14, cd24, cd157

## Abstract

Introduction

As per current guidelines, detection of paroxysmal nocturnal hematuria (PNH) clones on leucocytes requires the demonstration of the loss of at least two glycosyl-phosphatidyl-inositol (GPI)-linked molecules on both neutrophils and monocytes by flow cytometry. CD24 and CD14 are GPI-linked molecules expressed on neutrophils and monocytes respectively, whereas another GPI-linked molecule, CD157, is expressed on both neutrophils and monocytes. This prospective study evaluated the ability of CD157 to replace both CD24 and CD14 in a single-tube flow-cytometric assay to detect PNH clones on both neutrophils and monocytes.

Materials and methods

PNH clones were newly detected in 52 patients by an existing “standard” single-tube six-color flow-cytometric method, which was routinely performed in our laboratory at the time of undertaking this study. Six antibodies (CD45/CD15/CD64/CD24/CD14/FLAER) were used in this "standard" technique. Subjects were divided into two groups: (i) PNH disease (n=10), and (ii) aplastic anemia/myelodysplastic syndrome (AA/MDS) (n=42). Diagnosis of PNH disease and AA/MDS were made as per standard literature and guidelines. Results were compared with a single-tube five-color “test” assay using the antibodies CD45/CD15/CD64/CD157/FLAER by flow cytometry. Samples from 20 healthy control subjects were used to calculate cut-off values for the “test” assay.

Results

By the "test" method, cut-off values for detecting PNH clones obtained from receiver operating-characteristic curve analysis were >0.4% for neutrophils (sensitivity=96.15%, specificity=95%), and >0.9% for monocytes (sensitivity=98.08%, specificity=95%). There was significant correlation between PNH clone sizes measured by both the “standard” and “test” assays in neutrophils (PNH disease: r=0.976, p<0.001; AA/MDS: r=0.980, p<0.001) as well as monocytes (PNH disease: r=0.806, p=0.005; AA/MDS: r=0.915, p<0.001). Bland-Altman analysis showed agreement between both assays in all the 52 patients and in individuals with AA/MDS. The cost of the test to the patients was about 15% less in the “test” method than the ”standard” technique, with improved technical efficiency.

Conclusion

CD157 can replace both CD24 and CD14 in a single-tube flow-cytometric assay to detect PNH clones on both neutrophils and monocytes, with reduced cost to the patients and improved technical efficiency.

## Introduction

Paroxysmal nocturnal hemoglobinuria (PNH) is a rare, acquired clonal disorder of hematopoietic stem cells (HSCs), which clinically manifests as bone marrow failure, hemolytic anemia, smooth muscle dystonia, and thrombosis. The condition develops due to a somatic mutation of the phosphatidyl-inositol-glycan-class-A gene, leading to partial or complete deficiency of glycosyl-phosphatidyl-inositol (GPI) anchored proteins in the progeny of the affected HSCs [1]. Several laboratory tests are available for the diagnosis of PNH on a peripheral blood sample. These include the modified Ham’s test, sucrose lysis test, gel card technique, and flow cytometry. The latter is now the recommended method to detect PNH clones, based on the demonstration of the loss of at least two GPI-anchored proteins (GPI-APs) on red blood cells (RBCs), neutrophils, and monocytes in peripheral blood. Various guidelines have been published to standardize PNH testing by flow cytometry across laboratories; as per current recommendations, highly sensitive and specific flow-cytometric analysis on RBCs, neutrophils, and monocytes should be done simultaneously for patients suspected to harbor PNH clones [2-4].

CD157, a member of the CD38 supergene family, is among the GPI-APs recently studied for analysis of PNH clones on leucocytes. It is an ectoenzyme, which functions as an integrin receptor and signaling molecule. CD157 is expressed on both neutrophils and monocytes at a relatively high density, so it can obviate the need for the separate use of two different GPI-APs for neutrophils and monocytes (CD24 on neutrophils and CD14 on monocytes) in single-tube flow-cytometric techniques to detect PNH clones on leucocytes [5]. Another compound called aerolysin, a toxin secreted by the bacterium *Aeromonas hydrophilia*, is a channel-forming protein that binds selectively to GPI anchors. A fluorochrome-labeled modified recombinant form of aerolysin (that does not cause cell lysis) can be used to detect leucocytes with PNH phenotype [6]. This fluorescently labeled inactive variant of the protein aerolysin (FLAER) detects all GPI-APs, and so its primary advantage is that it can be used as a single and highly specific marker for PNH testing by flow cytometry on both neutrophils and monocytes. In addition, FLAER-based assays provide a better estimate of clonal size, particularly if PNH is associated with aplastic anemia (AA), myelodysplastic syndrome (MDS), or myeloproliferative neoplasms; they are now the techniques of choice to detect PNH clone on leucocytes [4]. The primary disadvantage is that FLAER does not bind well to RBCs and hence cannot be used to characterize GPI-AP expression on RBCs [1].

In the first published observation on the utility of CD157 in FLAER-based PNH assays on leucocytes by flow cytometry, Sutherland et al. analyzed several single-tube antibody cocktails, viz., four-color granulocyte assay using either predicate CD45/CD15/CD24/FLAER or CD45/CD15/CD157/FLAER combinations, four-color monocyte assay using either predicate CD45/CD64/CD14/FLAER or CD45/CD64/CD157/FLAER combinations, and a five-color technique using a combination of CD45/CD15/CD64/CD157/FLAER. Like the predicate four-color methods, the CD157-based four and five-color assays could satisfactorily delineate PNH leucocytes from leucocytes with normal GPI expression, with a high correlation between the clone sizes detected [5]. Thereafter, CD157 has been evaluated successfully in single-tube flow-cytometric techniques in several studies [7-12]. The reduction of one antibody in CD157/FLAER-based assays as compared to CD24/CD14/FLAER-based methods improves the technical efficiency of the laboratory and reduces the cost of the test to the patients [5,7-10].

We compared the efficacy of a CD157/FLAER-based single-tube “test” method to an existing “standard” CD24/CD14/FLAER-based technique that was used routinely in our laboratory to detect PNH clones on leucocytes by flow cytometry when this study was undertaken. Our aim was to evaluate the ability of CD157 to replace both CD24 and CD14 in a single-tube flow-cytometric assay in detecting PNH clones on both neutrophils and monocytes.

This study was previously presented as an oral paper (abstract) at the 58th annual national conference of the Indian Society of Hematology and Blood Transfusion (ISHBT) on November 3, 2017.

## Materials and methods

Study design

A prospective study was conducted in the Department of Hematology, All India Institute of Medical Sciences (AIIMS), New Delhi from November 2016 to June 2017.

Ethical approval and financial grant

Institute Ethics Committee of AIIMS, New Delhi for Postgraduate Research approved the study vide reference no. IECPG/14/28.10.2015, RT-35/27.01.2016, dated 15.02.2016. A financial grant to conduct the study was received in the year 2017 from Hematology Education and Research Society (HERS), AIIMS, New Delhi, and ISHBT (Indian rupees 34,308, received on 01.07.17, Reference No. 567913). The principles of the Declaration of Helsinki were applied throughout the study.

Consent

Written and informed consent was obtained from all the patients and control subjects who participated in the study.

Characteristics of patients and control subjects

Fifty-two patients who were newly detected to have PNH clones on both neutrophils and monocytes by a routinely performed “standard” flow-cytometric assay in our laboratory were prospectively analyzed, including two patients with neutrophil clone sizes below the cut-off used (details of these two subjects are provided in the “Results” section below, under the sub-section ‘’PNH clone sizes below the cut-off used’’). Patients under follow-up for previously detected PNH clones on leucocytes and/or RBCs (with or without associated AA/MDS) were excluded. Samples from 20 healthy age and sex-matched control subjects were used to establish cut-off values for the “test” method by receiver operating characteristic curve analysis. Subjects were classified into two groups: (i) PNH disease (n=10), (ii) AA/MDS with PNH clone (n= 42). PNH disease was diagnosed in patients with clinical features of the disease, with no preceding history of AA/MDS [1]. Diagnosis of AA and MDS were made according to established guidelines [13-15].

Sample collection

Three mL of peripheral blood anticoagulated with ethylene-diamine-tetra-acetic acid was collected and processed within four to six hours of sampling.

Protocol for flow cytometry (stain-lyse-wash)

A complete blood count was obtained with the Sysmex-XT-1800i hematology analyzer (Sysmex Corporation, Kobe, Japan). Fifty microlitres of whole blood were used per tube for both the “standard” and the “test” assays. Pre-titrated volumes of six antibodies (5µL CD45-PerCP-Cy5.5, 3µL CD15-APC, 3.5µL CD64-PE-Cy7, 3µL CD14-APC-Cy7, 5µL CD24-PE, and 5µL FLAER-FITC) were then added to the tube designed for the “standard” technique. For the “test’ method, instead of CD24 and CD14, 3 µL of CD157-PE was added, while the other antibodies remained the same as in the “standard” assay. All antibodies were manufactured by Becton Dickinson (BD, Franklin Lakes, NJ) except FLAER, which was procured from Cedarlane (Cedarlane Laboratories, Burlington, Canada). For negative control and autofluorescence, the same volume of sample in another tube was stained with only 5µL CD45-PerCP-Cy5.5. After adding antibodies, the tubes were incubated at room temperature for 30 minutes in the dark, followed by washing in sheath fluid. Thereafter, 2mL of BD-FACS-Lyse was added to each tube to lyse RBCs, and the tubes were incubated for 10 minutes at room temperature in dark. Each tube was then washed in sheath fluid twice and finally, 500µL of sheath fluid was used to suspend the cells for acquisition.

Acquisition and analysis

Cells were acquired on a BD-FACS-Canto-II^TM^ (six color/dual laser) flow cytometer. Standard methods recommended by the manufacturer were used to set up the instrument including photomultiplier tube voltages, threshold, compensation, and quality control. At least 10,000 events were acquired per tube. For samples with very low total leucocyte count, cells were acquired till the cell suspension was exhausted.

**Figure 1:**
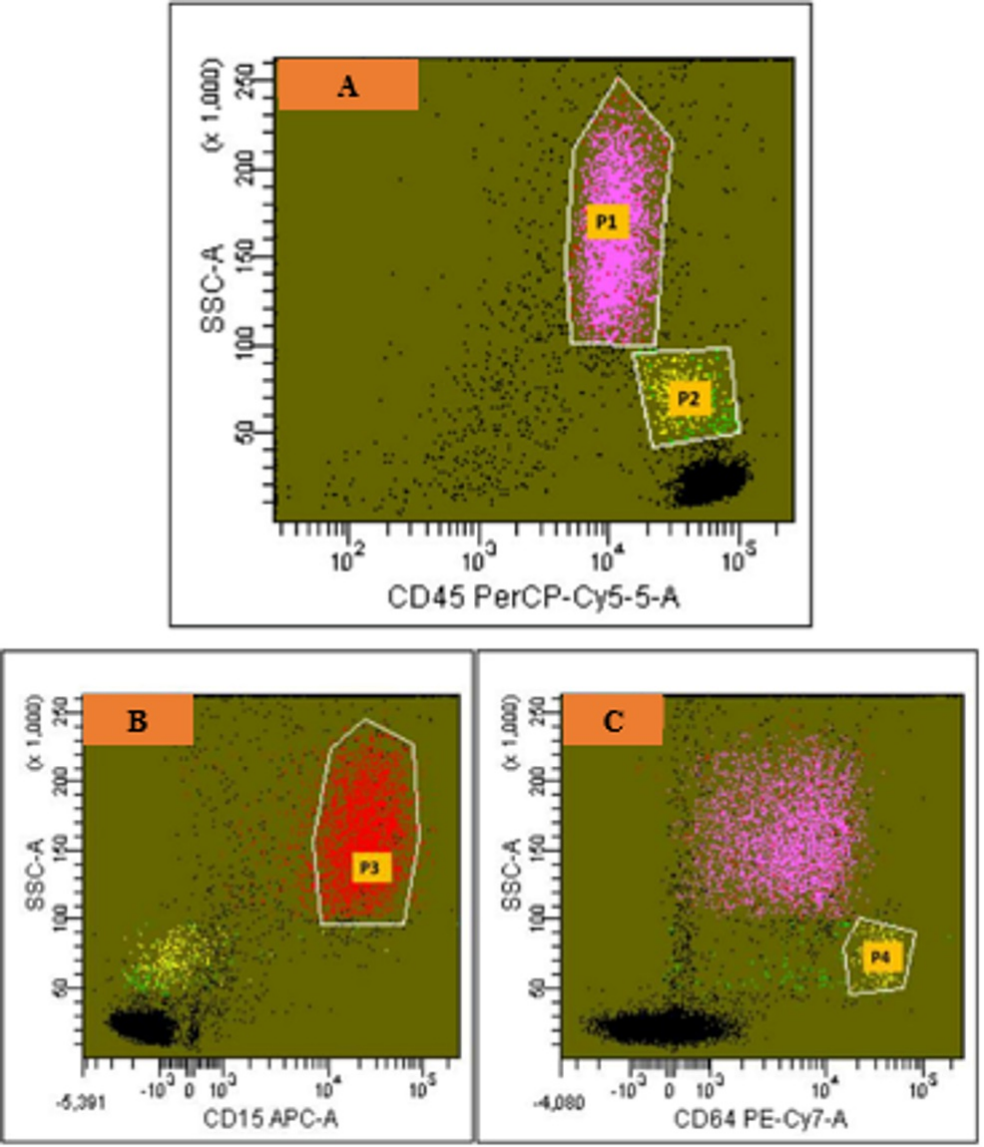
Flow cytometry on a peripheral blood sample for detecting PNH clones - lineage-gating strategies for leucocytes For neutrophils, initially, a broad gate P1 is put on CD45/SSC plot (A), followed by a lineage-specific gate P3 on CD15/SSC plot (B). For monocytes, an initial broad gate P2 is put on CD45/SSC plot (A), followed by a lineage-specific gate P4 on CD64/SSC plot (C). For determining PNH clone size, further analysis is done on the neutrophils gated on “P3” and monocytes gated on “P4” CD: cluster of differentiation; PNH: paroxysmal nocturnal hematuria; SSC: side scatter

**Figure 2:**
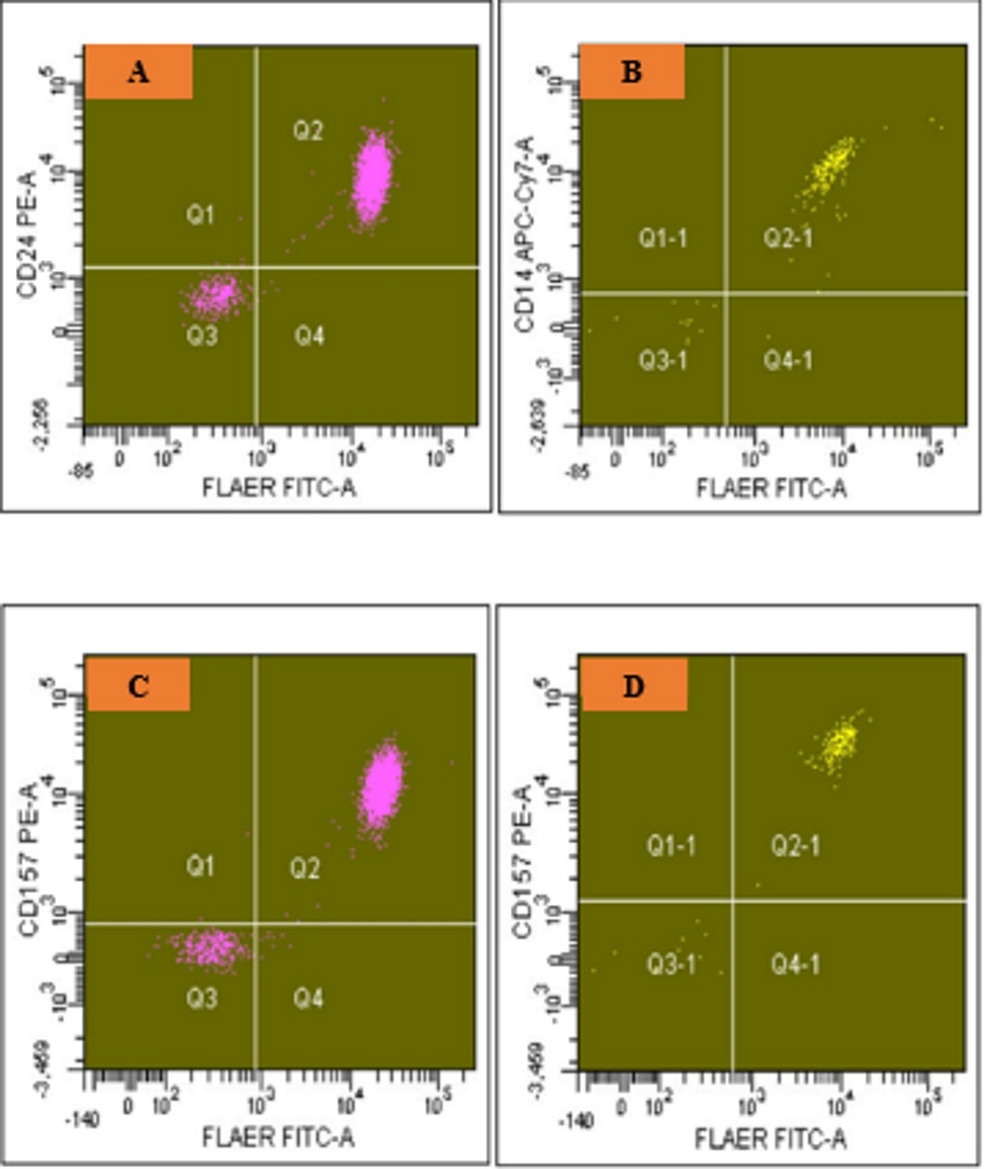
Flow cytometry on a peripheral blood sample showing PNH clones on neutrophils and monocytes after lineage gating as per Figure 1 For the “standard” method, CD24/FLAER double negative PNH neutrophils are detected in quadrant Q3 (9.9%) (A), whereas CD14/FLAER double negative PNH monocytes are detected in quadrant Q3-1 (5.5%) (B). For the “test” assay, CD157/FLAER double negative PNH neutrophils are detected in quadrant Q3 (10.1%) (C), whereas CD157/FLAER double negative PNH monocytes are detected in quadrant Q3-1 (5.6%) (D). Note: data and plots are from the same sample; the percentages mentioned above are not displayed in the figure PNH: paroxysmal nocturnal hematuria; CD: cluster of differentiation

BD-FACS-Diva^TM^ software was used for analysis. For both techniques, initially, two broad gates were applied for neutrophils (P1 gate) and monocytes (P2 gate) on CD45/SSC plot (Figure 1A), followed by lineage gating for neutrophils (P3 gate) on CD15/SSC plot (Figure 1B) and monocytes (P4 gate) on CD64/SSC plot (Figure 1C). For the “standard” assay, PNH clone size on the lineage-gated neutrophils was determined by the percentage of the double negative population for CD24/FLAER in quadrant Q3 (Figure 2A), whereas the percentage of the double negative population for CD14/FLAER in quadrant Q3-1 was considered as PNH clone on the lineage-gated monocytes (Figure 2B). Based on the results of a previous study carried out in our department, PNH clone sizes of >0.7% on neutrophils and >0.9% on monocytes were considered positive for the “standard” method [16]. Lineage-gated neutrophil and monocyte populations, which were double negative for CD157/FLAER (neutrophil population in quadrant Q3, Figure 2C, and monocyte population in quadrant Q3-1, Figure 2D), were used to quantify PNH clones for the “test” assay.

Statistical analysis

Data were analyzed by the Stata 14.0 software (StataCorp LP, College Station, TX). Qualitative data were expressed as frequencies and percentages. Quantitative data were expressed as median (min-max) as the data followed skewed distribution. Rank sum analysis was used to compare the median between the groups. The Chi-square test was used to compare proportions. Spearman’s correlation coefficient was used to find the correlation between parameters. Bland-Altman plot was used to assess agreement between two techniques (“standard” and “test” methods). A p-value <0.05 was considered statistically significant.

## Results

Out of the 52 patients analyzed, 10 (19%) were diagnosed to have PNH disease, and 42 (81%) individuals had PNH clones in association with AA/MDS.

Cut-off for detection of PNH clone by “test” method

**Figure 3:**
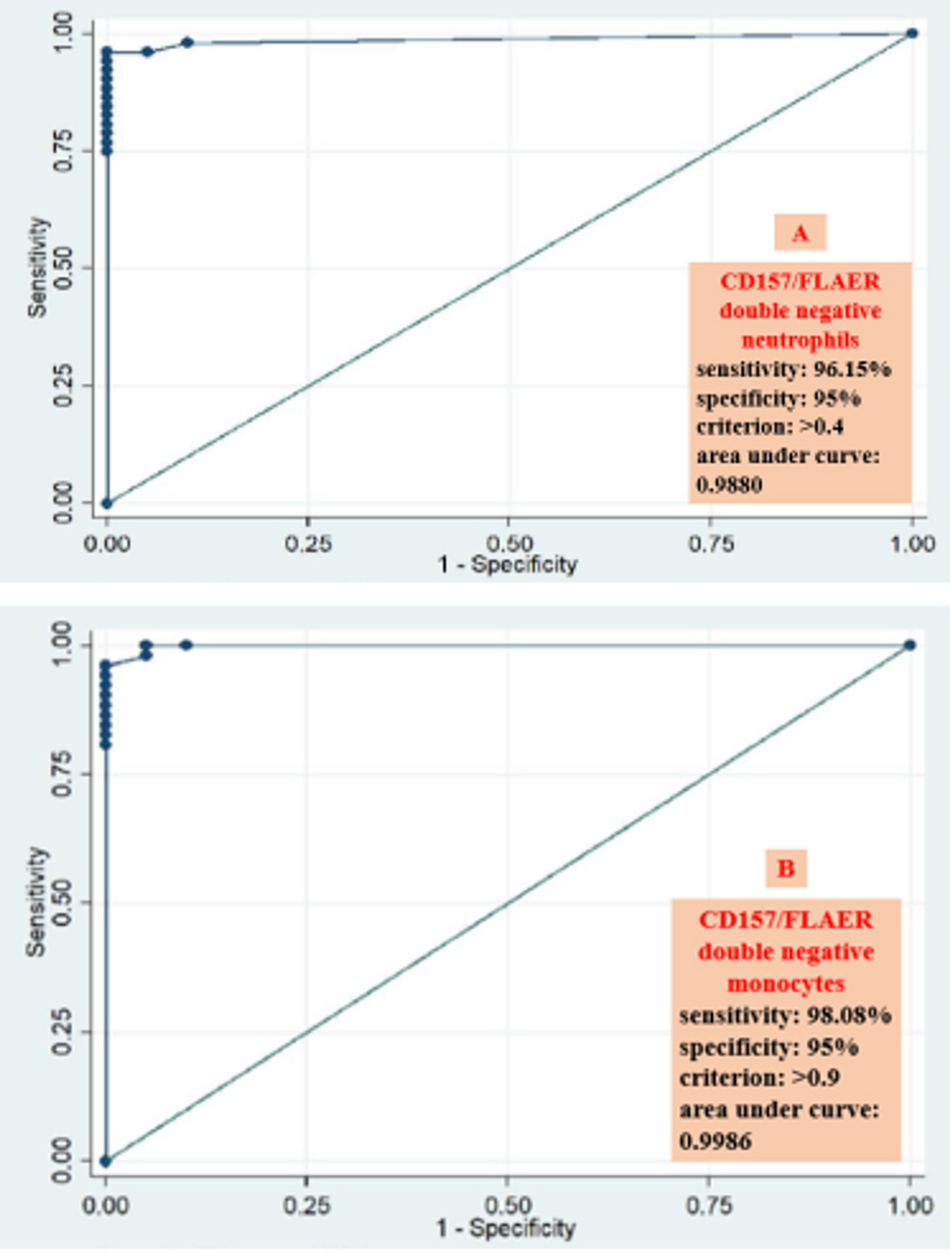
Receiver operating characteristic curve analysis for CD157/FLAER-based detection of PNH clone on neutrophils (A) and monocytes (B) CD: cluster of differentiation, PNH: paroxysmal nocturnal hematuria

Using receiver operating characteristic curve analysis, PNH clone size of >0.4% on neutrophils was considered positive (sensitivity=96.15%, specificity=95%, Figure 3A), whereas for monocytes, PNH clone size of >0.9% was considered positive (sensitivity=98.08% and specificity=95%, Figure 3B) for the “test” method.

PNH clone sizes measured by both “standard” and “test” methods in patients with PNH disease

By the “standard” and “test” methods, median neutrophil clone sizes detected in this group were 84% (42.8-97%) and 84.3% (42.4-94.7%) respectively; median monocyte clone sizes were found to be 90.1% (41.8-97.6%) and 89.9% (51.4-94.5%) by the two techniques respectively (Table 1).

**Table 1:** Clone sizes in individuals with PNH disease and in patients with AA/MDS having PNH clone Note: data represented as median (min-max) PNH: paroxysmal nocturnal hemoglobinuria; AA/MDS: aplastic anemia/myelodysplastic syndrome

Leucocyte subtype	Size of PNH clone in percentage (%) at diagnosis in patients with PNH disease (n=10)		Size of PNH clone in percentage (%) at diagnosis in patients of AA/MDS with PNH clone (n=42)	
	“Standard” method	“Test” method	“Standard” method	“Test” method
Neutrophils	84 (42.8-97)	84.3 (42.4-94.7)	9.4 (0-97.2)	10.9 (0-97.3)
Monocytes	90.1 (41.8-97.6)	89.9 (51.4-94.5)	11.4 (1.1-94.3)	11.9 (0.7-98)

PNH clone sizes measured by both “standard” and “test” methods in patients with AA/MDS having PNH clone

Median neutrophil clone sizes detected in these patients were 9.4% (0-97.2%) and 10.9% (0-97.3%) by the “standard” and “test” assays respectively; median monocyte clone sizes found were 11.4% (1.1-94.3%) and 11.9% (0.7-98%) by the two methods respectively (Table 1).

Correlation of CD157/FLAER with CD24/FLAER and CD14/FLAER in measuring PNH clone sizes in patients with PNH disease

**Figure 4:**
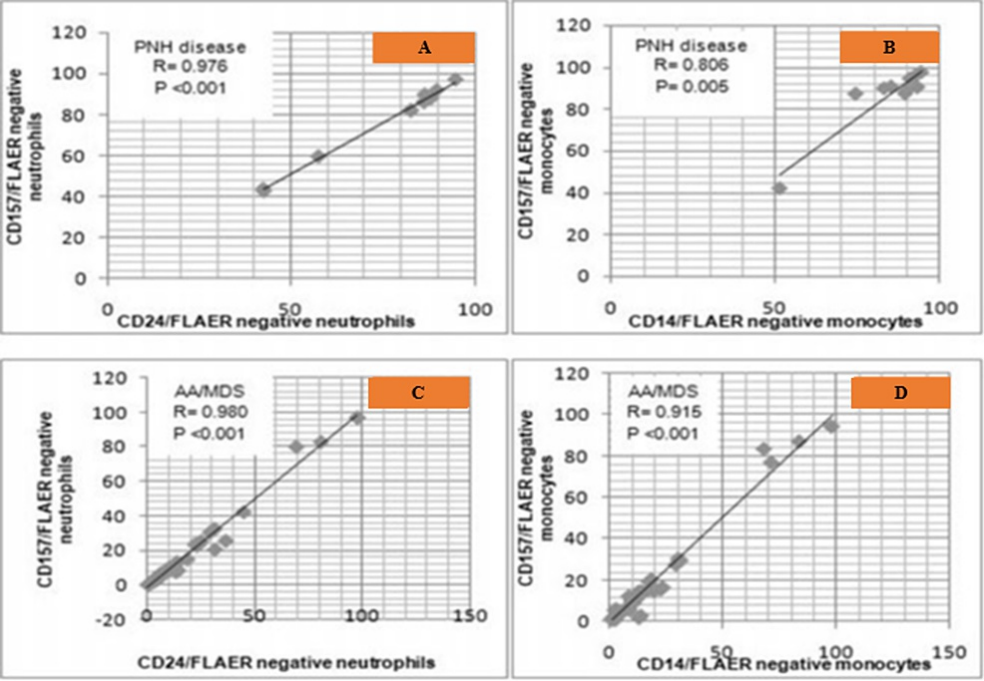
Correlation of PNH clone sizes measured on neutrophils and monocytes between the “test” method using CD157/FLAER, and the “standard” method using CD24/FLAER for neutrophils and CD14/FLAER for monocytes in patients with PNH disease (A, B) and in individuals with AA/MDS having PNH clone (C, D) CD: cluster of differentiation; PNH: paroxysmal nocturnal hemoglobinuria; AA/MDS: aplastic anemia/myelodysplastic syndrome; R: correlation coefficient

In this group, there was a significant correlation between the clone sizes measured by the “standard” and “test” assays on neutrophils with a correlation coefficient (r=0.976 and p<0.001 Figure 4A), as well as on monocytes (r=0.806, p=0.005, Figure 4B).

Correlation of CD157/FLAER with CD24/FLAER and CD14/FLAER in measuring PNH clone sizes in patients with AA/MDS having PNH clone

In these patients, there was a significant correlation between the clone sizes measured by the “standard” and “test” techniques on neutrophils (r=0.980 and p<0.001, Figure 4C), as well as monocytes (r=0.915, p<0.001, Figure 4D).

PNH clone sizes below the cut-off used

In one patient with AA/MDS, neutrophil clone sizes detected were below the cut-off used: the sample had a neutrophil clone size of 0.2% measured by the “standard” method and 0.1% detected by the “test” assay, whereas monocyte clone sizes were found to be 1.9% and 0.9% by the two techniques respectively. In another patient with AA/MDS, neutrophil clones were not picked up by both assays (0%); whereas monocyte clone sizes detected were 1.7% by the “standard” method and 1.1% by the “test” technique.

Bland-Altman analysis for agreement between “standard” and “test” assays

**Figure 5:**
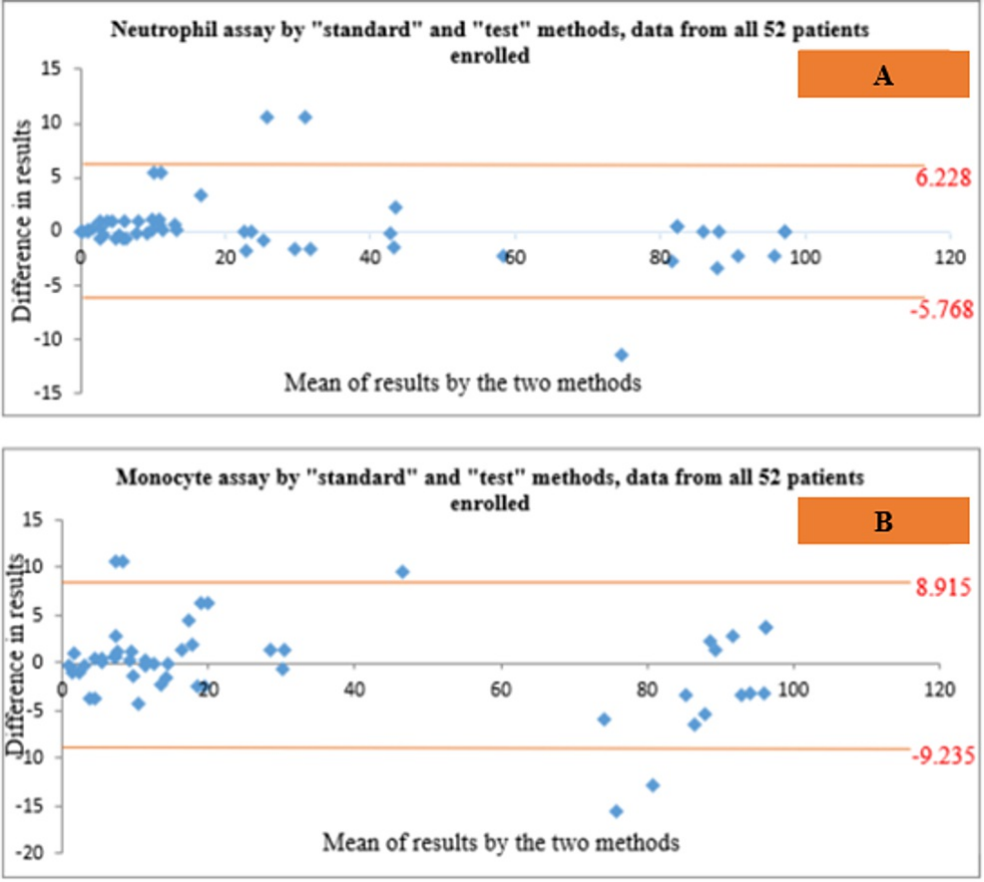
Bland-Altman plots showing agreement between the “standard” and “test” methods in the detection of PNH clones on neutrophils (A) as well as monocytes (B) in all 52 patients enrolled in the study PNH: paroxysmal nocturnal hemoglobinuria

**Figure 6:**
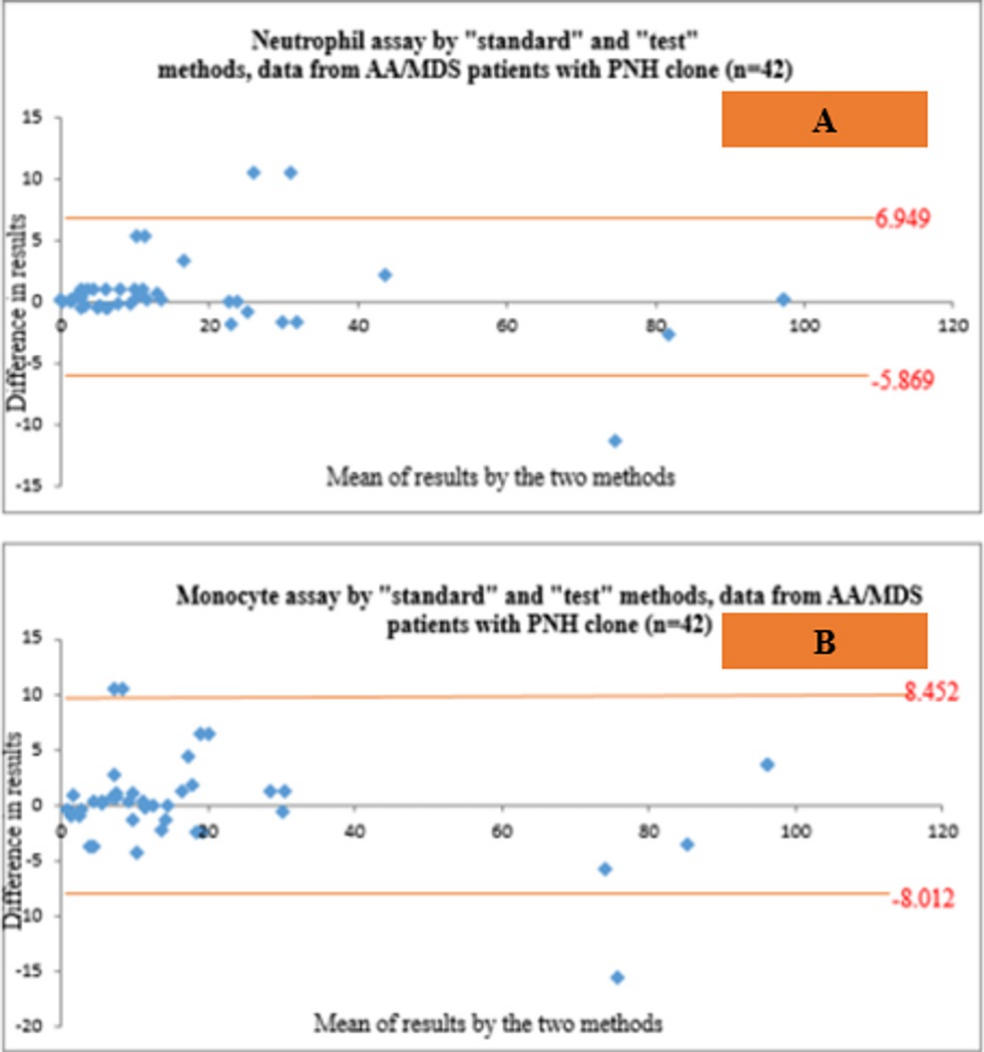
Bland-Altman plots showing agreement between the “standard” and the “test” methods in the detection of PNH clones on neutrophils (A) as well as monocytes (B) in AA/MDS patients with PNH clone (n=42) AA/MDS: aplastic anemia/myelodysplastic syndrome, PNH: paroxysmal nocturnal hemoglobinuria

Bland-Altman analysis showed agreement between both the assays in the detection of PNH clones on neutrophils and monocytes in all the 52 patients included (Figures 5A, 5B) as well as in the 42 individuals with AA/MDS (Figures 6A, 6B).

Comparison of cost-benefit analysis and technical considerations

The “standard” and the “test” methods used six and five antibodies respectively. So, the “test” assay could reduce sample preparation time, with improved technical efficiency. However, these observations were not objectively quantified. The cost of the test to the patients was about 15% less with the “test” method than the “standard’ assay.

## Discussion

We compared two single-tube flow-cytometric assays for detecting PNH clones on neutrophils and monocytes in patients with PNH disease and those with AA/MDS having PNH clones. Our aim was to evaluate whether CD157 in a five-color “test” method can replace both CD24 and CD14 used in the existing “standard” six-color assay that was performed routinely in our laboratory. CD45, CD15, CD64, and FLAER were the common antibodies used in both techniques.

Optimal cut-offs for detection of PNH clones were derived to be >0.4% for neutrophils and >0.9% for monocytes in the “test” assay, which were similar to the “standard” technique (cut-off >0.7% for neutrophils, >0.9% for monocytes) established earlier in our laboratory [16]. Peghini and Fehr compared the expression of FLAER with CD55/CD59/CD16/CD66b/CD24/CD14 on leucocytes in patients with PNH and normal control [17]. Using FLAER, their assay could detect PNH clones as low as 0.48% on granulocytes, identical to the cut-off derived for neutrophils by the CD157/FLAER-based technique adopted by us. Borowitz et al. have defined routine PNH assays as those with a sensitivity of 1%, and high-sensitivity assays as the ones capable of detecting much smaller clones (at least 0.01%) [2]. More recent consensus guidelines have discussed detailed protocols including instrument set-up, reagent selection, assay validation, data analysis, and quality assurance for high-sensitivity PNH assays [4,18-19]. Our assay was not designed as a high-sensitivity PNH leucocyte analysis; however, the results of the study paved the way for establishing high sensitivity PNH assay for leucocytes using the CD157/FLAER based five-color method in the future.

Leucocytes processed by the stain-lyse-wash technique show better light scatter characteristics; however, leucopenic samples may be pre-lysed with ammonium chloride [2]. Using a stain-lyse-wash technique, the “test” method in our study could detect a wide range of PNH clones on leucocytes, viz., median neutrophil clones of 84.3% (42.4-94.7%) and 10.9% (0-97.3%) in patients with PNH disease and in individuals with AA/MDS respectively; median monocyte clone size in the two groups of subjects were 89.9% (51.4-94.5%) and 11.9% (0.7-98%) respectively. We found significant correlation between the PNH clone sizes measured by both the five and six-color methods on neutrophils (PNH disease: r=0.976, p<0.001; AA/MDS: r=0.980, p<0.001) and monocytes (PNH disease: r=0.806, p=0.005; AA/MDS: r=0.915, p<0.001).

Identical to our study, Marinov et al. compared the performance of a six-color standard assay using CD45/CD15/CD64/CD24/CD14/FLAER with a five-color test method using CD45/CD15/CD64/CD157/FLAER in the detection of PNH clones on leucocytes by flow cytometry. The investigators found a high correlation (r>0.99) between the two techniques for PNH clones ranging from 0.3 to 99.8% [7]. Rahman et al. have also reported a high correlation between two methods using the same antibodies (r>0.993) over a wide range of clone sizes (0.11-98.7%) [10]. Similar observations with a high degree of correlation between CD157/FLAER-based assays and CD24/CD14/FLAER-based techniques were observed by Sutherland et al. (r>0.99) over a wide range of PNH clones (0.06%-99.8%), and Galtseva et al. (r=0.9994 on granulocytes, r=0.9924 on monocytes) [5,8]. Another CD157/FLAER-based single-tube assay evaluated by Correia RP and co-workers could detect neutrophil and monocyte PNH clones of 0.2-76.0% and 0.9-77.0% respectively [9]. These observations support the ability of CD157/FLAER-based single-tube assays to detect a wide range of PNH clones on both neutrophils and monocytes with acceptable correlation to the methods based on CD24, CD14, and FLAER. Of note, our technique was not designed as a high-sensitivity PNH leucocyte assay, and hence we might have missed smaller clones measured by the methods adopted by other observers [5,7-12].

Bland-Altman analysis showed agreement between both the “standard” and “test” techniques in the detection of PNH clones in all the 52 individuals included, and in the 42 patients with AA/MDS having PNH clones, similar to the findings reported by Marinov et al. [7]. The agreement could not be represented in the subjects with PNH disease in our study, possibly due to having very few patients (n=10) in this group.

Type I, type II, and type III PNH populations were not separately evaluated in our analysis. Rahman et al. acquired a minimum of 1,00,000 neutrophils and found better separation between the normal population and PNH clone by the CD157/FLAER-based assay than the CD24/CD14/FLAER-based technique; type II populations were separated from type III populations more clearly [10]. In contrast, Marinov et al. acquired 50,000 granulocytes and did not observe better delineation of type II and type III granulocytes and monocytes with the CD157/FLAER-based approach compared to the CD24/CD24/FLAER-based analysis [7]. Whether acquiring more cells improves visual delineation between PNH-population types by increasing the number of events recorded in each type needs to be evaluated further.

Significant advantages of the five-color approach using CD157/FLAER have been highlighted by various authors, viz., the ability to detect both PNH neutrophils and monocytes in a single-tube assay, thereby reducing the time for sample preparation and analysis by the laboratory, with significant improvements in technical efficiency at a reduced cost of the test to the patients [5,7-10]. We noted a nearly 15% reduction of the cost per test to the patients by the CD157-based assay as compared to the method using both CD24 and CD14; a reduction of 10-15% in antibody cost was also observed by Rahman et al. [10]. Using activity-based costing, 12.7% lower cost was found in a CD157/FLAER-based five-color experiment compared to a four-color assay by Correia et al. [9].

For confirmatory evidence of the presence of PNH clones in a peripheral blood sample, deficiency of at least two GPI-APs should be demonstrated in at least two different types of cells out of neutrophils, monocytes, and RBCs [3]; more recent guidelines recommend simultaneous testing of RBCs, neutrophils, and monocytes by flow cytometry to detect GPI-AP deficiency [4]. In two patients with AA/MDS in our analysis, neutrophil clone sizes measured were below the cut-off used: neutrophil clones of 0.2% and 0.1% were detected in one of them with the “standard” and “test” methods respectively, with respective monocytes clones of 1.9% and 0.9%. In the other individual, neutrophil clones were not detected by both techniques (0%), whereas monocyte clones picked up were 1.7% by the “standard” and 1.1% by the “test” assays. In these two patients, the study of GPI-AP expression on RBCs could have been beneficial in further confirming the presence of PNH clones. This observation emphasizes the importance of analyzing PNH clones on RBCs in addition to neutrophils and monocytes by flow cytometry.

Naim et al. utilized the CD157-based five-color technique in patients with MDS; they reported that a cut-off for serum lactate dehydrogenase (LDH) of 247 IU can predict a PNH clone of >1% in these patients [11]. Their observation can help in judiciously prescribing PNH assays to individuals with MDS in resource-constrained settings. In another newer approach, a single-tube assay using nine antibodies (CD45/CD15/CD64/CD24/CD14/CD157/FLAER, with CD5/CD19 in the same fluorochrome for a "dump" channel to exclude non-specific binding) achieved a sensitivity of 0.01% for granulocytes and 0.05% for monocytes; the background on a normal whole blood sample was 0.00277% on granulocytes and 0.00076% on monocytes [12]. These recently published data offer scope for further clinically meaningful research using CD157/FLAER-based PNH leucocyte assays.

## Conclusions

Though our study was not designed as a high-sensitivity PNH leucocyte assay and had a small sample size (particularly in the group of patients with PNH disease), the CD157/FLAER-based single-tube five-color flow-cytometric approach could detect PNH clone sizes of <1% on both neutrophils and monocytes. The results were valid across a wide range of clone sizes in individuals with PNH disease and AA/MDS patients with PNH clones. The CD157/FLAER-based assay was found to be equivalent to the routinely used single tube CD24/CD14/FLAER-based six-color flow-cytometric technique in detecting PNH clones on leucocytes, with a reduction in the cost of the test to the patients by about 15%. Moreover, sample preparation time was reduced, providing better use of technical resources. This is particularly important in a high-volume laboratory like the one in our institute, which receives multiple samples for flow cytometry for a wide variety of malignant as well as benign hematological disorders daily. Further improvement in PNH testing will enable the establishment of high-sensitivity assays for RBCs, neutrophils, and monocytes as per recommendations by current guidelines.
